# A homology model of restriction endonuclease SfiI in complex with DNA

**DOI:** 10.1186/1472-6807-5-2

**Published:** 2005-01-24

**Authors:** Agnieszka A Chmiel, Janusz M Bujnicki, Krzysztof J Skowronek

**Affiliations:** 1 Laboratory of Bioinformatics and Protein Engineering, International Institute of Molecular and Cell Biology, Trojdena 4, 02-109 Warsaw, Poland

## Abstract

**Background:**

Restriction enzymes (REases) are commercial reagents commonly used in recombinant DNA technologies. They are attractive models for studying protein-DNA interactions and valuable targets for protein engineering. They are, however, extremely divergent: the amino acid sequence of a typical REase usually shows no detectable similarities to any other proteins, with rare exceptions of other REases that recognize identical or very similar sequences. From structural analyses and bioinformatics studies it has been learned that some REases belong to at least four unrelated and structurally distinct superfamilies of nucleases, PD-DxK, PLD, HNH, and GIY-YIG. Hence, they are extremely hard targets for structure prediction and homology-based inference of sequence-function relationships and the great majority of REases remain structurally and evolutionarily unclassified.

**Results:**

SfiI is a REase which recognizes the interrupted palindromic sequence 5'GGCCNNNN^NGGCC3' and generates 3 nt long 3' overhangs upon cleavage. SfiI is an archetypal Type IIF enzyme, which functions as a tetramer and cleaves two copies of the recognition site in a concerted manner. Its sequence shows no similarity to other proteins and nothing is known about the localization of its active site or residues important for oligomerization. Using the threading approach for protein fold-recognition, we identified a remote relationship between SfiI and BglI, a dimeric Type IIP restriction enzyme from the PD-DxK superfamily of nucleases, which recognizes the 5'GCCNNNN^NGGC3' sequence and whose structure in complex with the substrate DNA is available. We constructed a homology model of SfiI in complex with its target sequence and used it to predict residues important for dimerization, tetramerization, DNA binding and catalysis.

**Conclusions:**

The bioinformatics analysis suggest that SfiI, a Type IIF enzyme, is more closely related to BglI, an "orthodox" Type IIP restriction enzyme, than to any other REase, including other Type IIF REases with known structures, such as NgoMIV. NgoMIV and BglI belong to two different, very remotely related branches of the PD-DxK superfamily: the α-class (EcoRI-like), and the β-class (EcoRV-like), respectively. Thus, our analysis provides evidence that the ability to tetramerize and cut the two DNA sequences in a concerted manner was developed independently at least two times in the evolution of the PD-DxK superfamily of REases. The model of SfiI will also serve as a convenient platform for further experimental analyses.

## Background

Type II restriction endonucleases (REases) comprise one of the major families of endonucleases and one of the largest groups of experimentally characterized enzymes (comprehensively reviewed in: [1]). The "orthodox" Type IIP REases are dimeric, they recognize a short (4–8 bp) palindromic sequence of double-stranded DNA and in the presence of Mg^2+^, catalyze the hydrolysis of phosphodiester bonds at precise positions within or close to this sequence, leaving "blunt" or "sticky" ends (with a 5' or 3' overhangs). The enzymes that do not fit this definition or exhibit certain structural and functional peculiarities, have been classified into several subtypes (review: [2]). REases coupled with DNA methyltransferases (MTases) of similar specificity form restriction-modification (RM) systems, which are ubiquitous among Bacteria and Archaea [3]. While cleavage at specific sequences provides efficient means of destroying foreign DNA, methylation of these sequences in the prokaryotic chromosome renders them resistant to REase and thereby protects the own DNA from cleavage. Because cleavage of the chromosomal DNA in unmodified sequences would be deletorious for the cell, the REases must maintain extremely high specificities, tightly coupled with that of the methyltransferase. A change in just one base pair of the "cognate" site can reduce the ratio *k*_cat_/*K*_m _for DNA cleavage by a factor ≥10^6 ^[4].

To date, only crystal structures of 15 REases have been solved, compared to over 3000 biochemically characterized enzymes (review: [1]; see also [3] for updates). It was found that they share a characteristic structural core and a very weakly conserved catalytic motif (P)D-X_n_-(D/E)-X-K (where X is any amino acid), together with a number of non-specific and structure-specific nucleases, suggesting that these proteins are evolutionarily related despite the absence of overall sequence similarity (review: [5]). The comparison of crystal structures of members of this so-called "PD-DxK" superfamily suggested that the catalytic and DNA-binding regions are major determinants of structural stability of these proteins [6]. Structural comparisons revealed also two major branches or classes, α and β, whose archetypal members were the enzymes that cleave DNA to generate 4 nt long 5' "sticky ends", for instance EcoRI (α-class), and those that generate "blunt" ends after the cleavage, for instance EcoRV (β-class) [7-9]. Using the experimentally solved structures as templates, bioinformatics methods such as iterative sequence database searches and protein fold-recognition have been used to predict the active site in some REases [10-13]. From these analyses it was learned that certain functional peculiarities, like the requirement of a binding of an uncleaved effector site for cleavage of another site characteristic for Type IIE enzymes, evolved independently in the α and β branches of the PD-DxK superfamily [11,13-15].

It was also found that other REases belong to completely unrelated superfamilies, with different three-dimensional folds and catalytic sites (review: [9]): BfiI is a member of the phospholipase D (PLD) superfamily [16], Eco29kI belongs to the GIY-YIG superfamily [17], and KpnI and a few other REases belong to the HNH superfamily [17-19]. While the structural information is essential to infer the molecular basis of sequence specificity in REases, the lack of overall sequence conservation in these enzymes, the absence of invariable residues even in the active site and the presence of several alternative folds make structure prediction and classification extremely difficult.

SfiI is a REase isolated from *Streptomyces fimbriatus*. It recognizes the interrupted palindromic sequence 5'GGCCNNNN^NGGCC3', where N denotes any base, and cleaves it as indicated by "^", leaving 3' extensions 3 nt long [20]. SfiI is a prototype of Type IIF enzymes, which function as tetramers that bind simultaneously to two recognition sites and cleave both sites concertedly [21]. However, a structural model of SfiI, which could be used as a platform to study its sequence-function relationships, is not yet available. Thus, despite the availability of a large body of biochemical data on how SfiI interacts with the DNA substrate (mainly on the kinetics of protein-DNA interactions with different substrates and the geometry of DNA looping [22-25], but not on the "residue-level" details thereof), the identity of amino acid residues important for dimerization, tetramerization, DNA binding and cleavage remains completely unknown. We have therefore carried out bioinformatics analyses of SfiI that allowed to identify its closest relative amongst REases with known structure and use this information to construct a tertiary model of SfiI in complex with its target DNA.

## Results and discussion

In the absence of experimentally determined protein structures, homology-based models may serve as working models for the investigation of sequence-structure-function relationships between diverged enzymes [26]. Homology-modeled structures may be of too low resolution to characterize the protein-protein or protein-DNA contacts at the atomic level, but they can suggest which sequence regions or individual amino-acids are essential components of the binding surfaces. In particular, identification of amino acids potentially involved in protein-DNA contacts may guide mutagenesis experiments aimed at the engineering protein variants with novel specificities. However, homology modeling requires a homologous template structure to be identified and the sequence of the protein of interest (a target) to be correctly aligned to the template.

### Identification of the three-dimensional fold of SfiI

The sequence of SfiI showed no significant similarity to any other protein sequences. Also among the proteins reported by BLAST with sub-optimal scores, there were no proteins of known structure and no nucleases (data not shown) that could hint at potential relationships of SfiI to any previously characterized protein superfamily. Thus, in order to identify a template structure for modeling of SfI we used the threading approach, which allows to assess the compatibility of the target sequence with the available protein folds based not only on the sequence similarity but also on the structural considerations (match of secondary structure elements, compatibility of residue-residue contacts, etc.) (reviews: [27,28]). The SfiI sequence was therefore submitted to the GeneSilico protein fold-recognition metaserver [29]. As expected, fold-recognition methods that rely only on sequence similarity (PDB-BLAST, and FFAS) failed to identify any significant matches between SfiI and proteins with known structrues. However, several threading methods that explicitly use the structural information from the templates reported a match between SfiI and the structure of a Type II REase BglI [30], a member of the PD-DxK superfamily of nucleases (FUGUE [31]: 4.25, INBGU [32]: 3.8, SAM-T02 [33]: 0.13, 3DPSSM [34]: 4.4; note that these scores are not normalized as each server uses a different evaluation system; see the individual references for details). Additionally, FUGUE reported a match (low score 3.17) between SfiI and the structure of another REase, EcoRV [35]. Despite the scores reported by the individual threading methods (except FUGUE for BglI) were hardly significant, the consensus server Pcons5 [36] assigned a significant score (1.35) to the BglI structure as a potential modeling template.

### Homology modeling of the SfiI monomer

**Figure 1:**
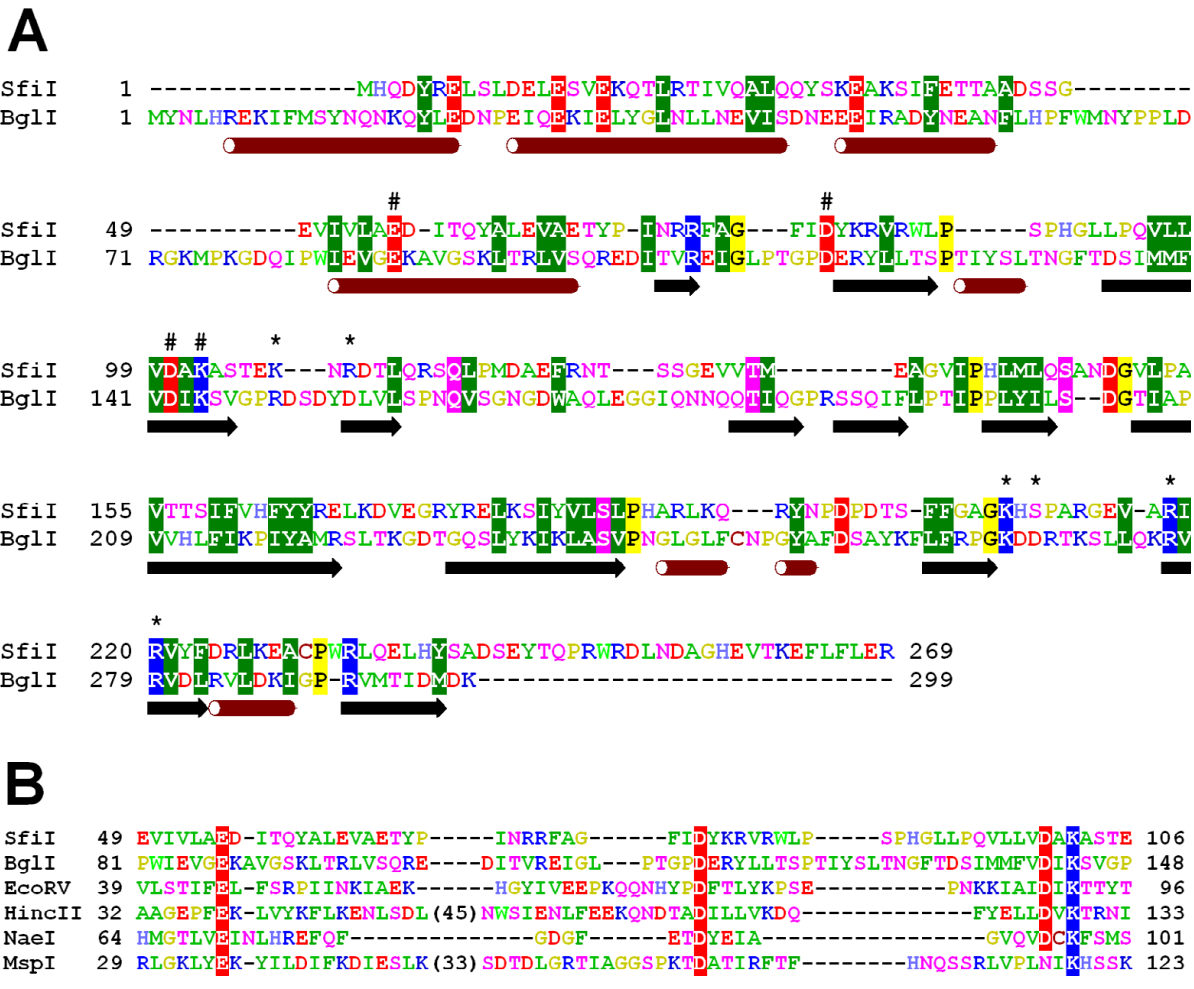
**Alignment between SfiI and structurally characterized REases. **A) Fold-recognition alignment between full-length sequences of SfiI and BglI. Amino acids are colored according to the physico-chemical properties of their side-chains (negatively charged: red, positively charged: blue, polar: magenta, hydrophobic: green. Pairs of residues conserved between SfiI and BglI are highlighted. Putative catalytic residues are indicated by "#", putative DNA-binding residues are indicated by "*". Secondary structure elements of BglI are shown below the alignment. Numbers of amino acid residues at the N-terminus of each panel are shown. B) Structure-based sequence alignment of the conserved core, corresponding to the PD-(D/E)XK motif, including SfiI, BglI, and other selected REases from the β-class. Conserved residues of the active site are highlighted.

**Figure 2:**
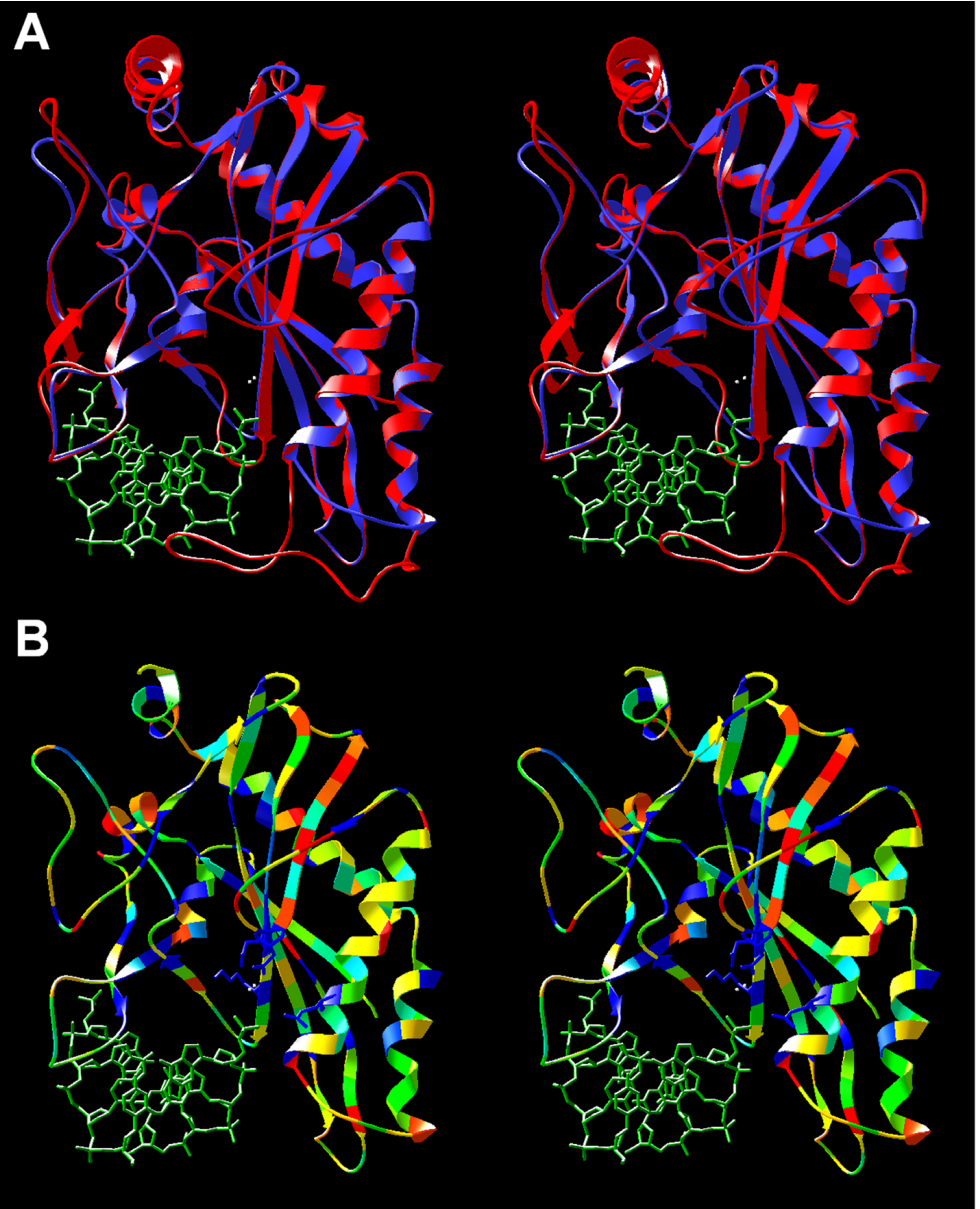
**Homology model of the SfiI monomer. **A) Superposition of the BglI template structure (red) and the SfiI model (blue). The CCGG half-site of the DNA target is shown in green. The Ca^2+ ^ions from the BglI structure are shown as white dots. B) SfiI model colored according to the sequence conservation: residues identical between SfiI and BglI are shown in blue, residues with physico-chemically similar side chains are in green, dissimilar residues are in yellow and red. The putative conserved active site is shown in the wireframe representation.

A homology model of SfiI was constructed based on the alignments reported by threading methods, using the "FRankenstein's Monster" approach [37] (see Methods). Since the PD-DxK nuclease fold was selected by Pcons as the only reasonable template and no other nuclease folds were identified by the FR methods, only alignments between SfiI and the PD-DxK superfamily members BglI and EcoRV were used. The final model was constructed by iterating the homology modeling procedure (initially based on the raw FR alignments), evaluation of the sequence-structure fit by VERIFY3D, merging of fragments with best scores, and local realignment in poorly scored regions. Local realignments were constrained to maintain the overlap between the secondary structure elements found in the bglI structure used as the modeling template, and predicted for SfiI. This procedure was stopped when all regions in the protein core obtained acceptable VERIFY3D score (>0.3) or their score could not be improved by any manipulations, while the average VERIFY3D score for the whole model could not be improved. The final model, comprising residues 13–240 obtained the average VERIFY3D score of 2.6. The alignment between SfiI and BglI is shown in Figure 1, the corresponding final model of the monomer is shown in Figure 2.

### Modeling of the SfiI dimer in complex with the DNA

**Figure 3:**
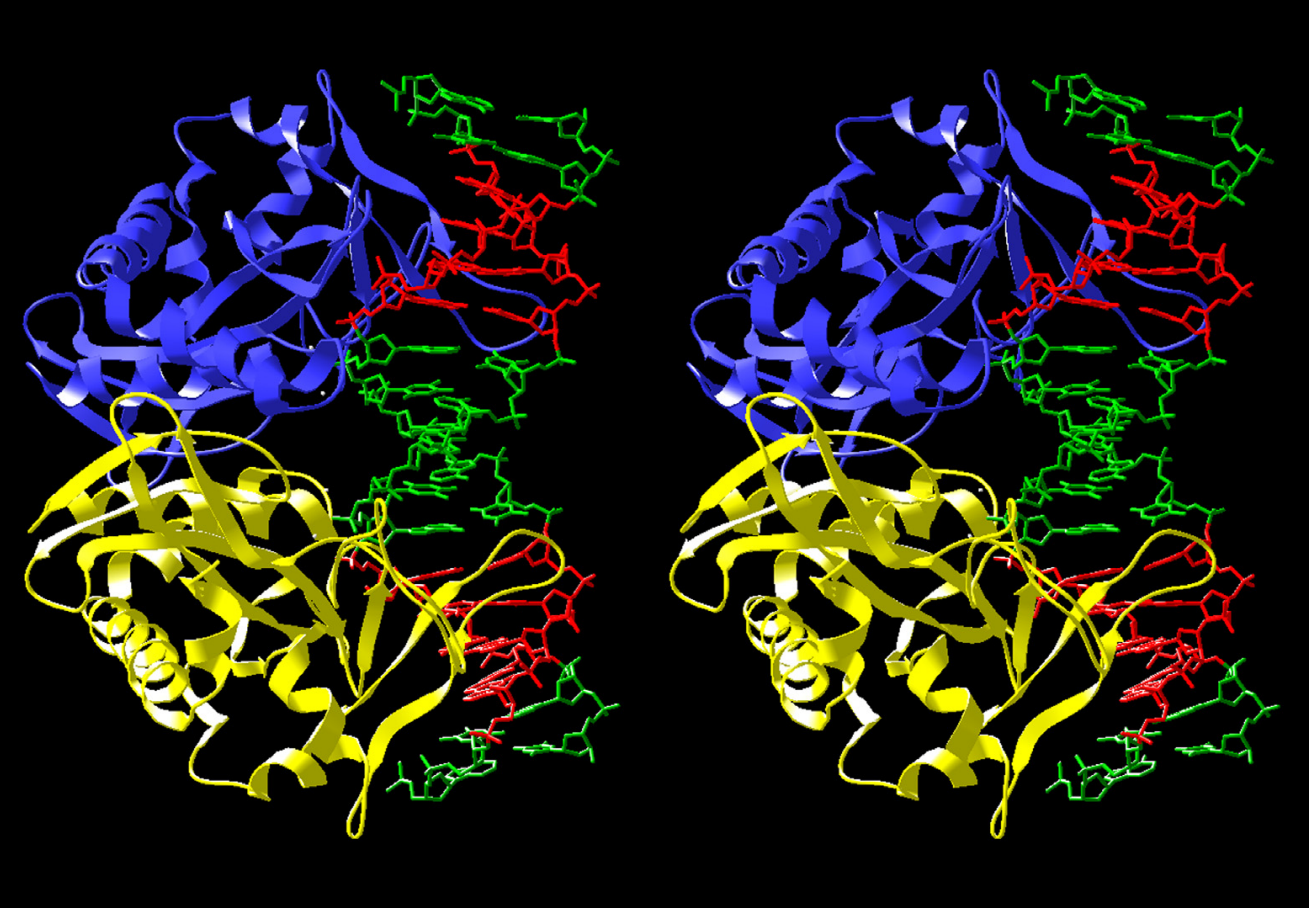
**Model of the SfiI dimer. **Individual subunits are shown in yellow and blue. The modeled DNA sequence is shown in green, the specifically recognized CCGG half-sites are in red.

BglI belongs to the "EcoRV-like" β-class of PD-DxK nucleases. The most typical features of REases from this class are: antiparallel orientation of the 5^th ^strand of the common β-sheet and recognition of the DNA by an additional β-sheet formed by extended loops between the common secondary structure elements [5,7,9]. Most of β-class PD-DxK REases (including EcoRV) exhibit a similar mode of dimerization, which results in positioning of the two active sites as to cut the pair of the opposite phosphodiester bonds in the middle of the recognition sequence and thereby produce the "blunt" ends. BglI is exceptional in that its mode of dimerization is completely different, which leads to a different arrangement of the active sites and the sequence-recognition loops, resulting in the recognition of an interrupted sequence 5'GCCNNNN^NGGC3' and cleavage in the position indicated by "^" that yields 3' ends 3 nt long. SfiI also recognizes an interrupted sequence 5'GGCCNNNN^NGGCC3' and cleaves it in the same manner and therefore can be regarded as a more specific variant of BglI. These striking functional similarities, together with the results of the threading analysis, suggest that SfiI is indeed closely related to BglI and that both enzymes interact with their substrate DNA in a similar manner. Thus, we modeled the structure of the SfiI dimer in complex with the DNA based on the available crystal structure of BglI [30]. Briefly, the SfiI monomer model was duplicated and each of the copies was superimposed onto the corresponding monomer in the BglI dimer. A few minor steric clashes between the side-chains of residues at the protein-protein interface were removed by choosing alternative rotamers for the respective amino acids. The DNA duplex (sequence 5'ATCGCCTAATAGGCGAT3') was copied from the BglI co-crystal structure (1 dmu) [30] and "mutated" to 5'ATGGCCTAATAGGCCAT3' using HyperChem 7.1 (Hypercube, Inc.), followed by local geometry optimization. One of the adenine residues in the mismatched A/A base pair in the middle of the DNA molecule was "mutated" to T/A. The curvature of the DNA remained unchanged. Essentially, the global structure of the protein-DNA complex for SfiI remains exactly as in the BglI structure, as reliable modeling of macromolecular interactions remains beyond the capabilities of the existing methods. The model of SfiI dimer is shown in Figure 3 and is available for download from .

### Model-based identification of amino acid residues important for catalysis, DNA-binding and dimerization of SfiI

**Figure 4:**
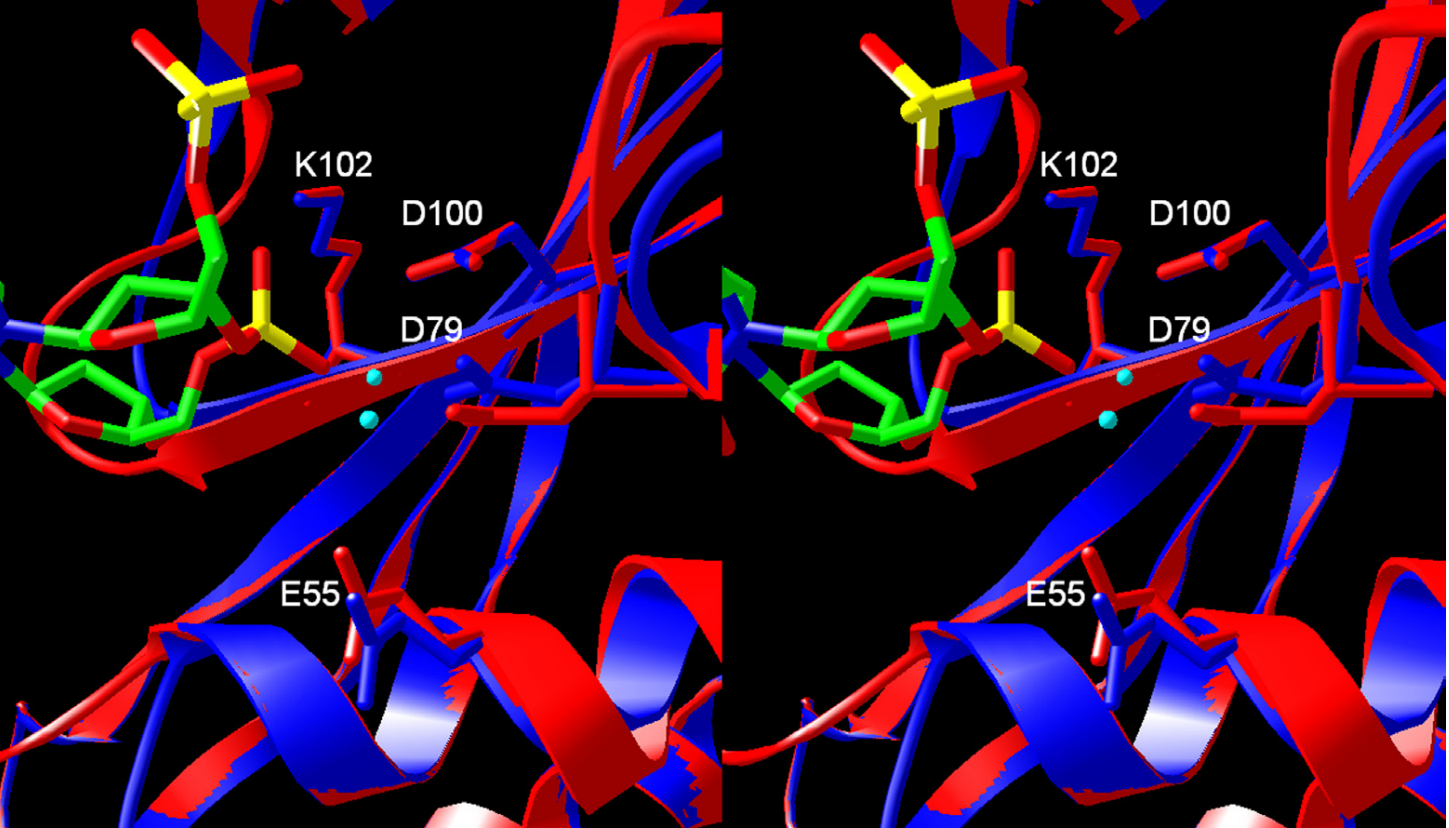
**Superposition of SfiI and BglI structures. **The predicted active site of SfiI (in blue) superimposed onto the BglI structure (red) Individual subunits are shown in yellow and blue. The Ca^2+ ^ions from the BglI structure are shown as cyan spheres. Only the two nucleotides adjacent to the scissile phosphodiester bond are shown.

**Figure 5:**
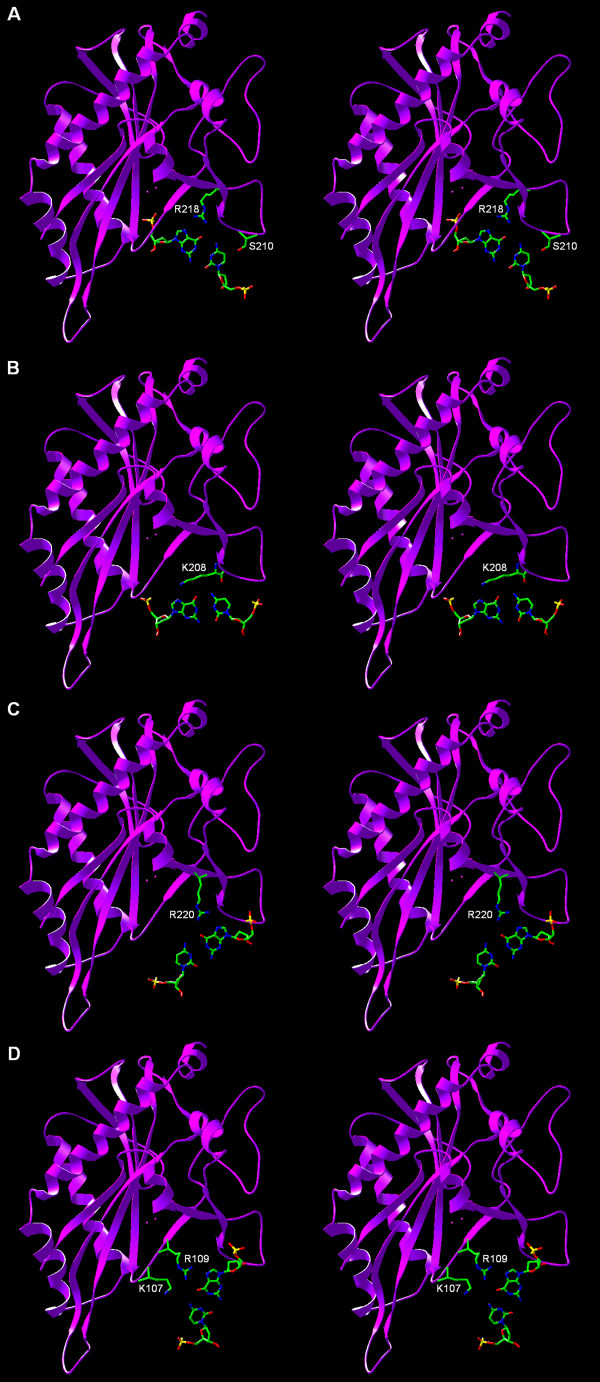
**Predicted specific protein-DNA contacts. **A) Recognition of the inner C/G pair B) Recognition of the middle C/G pair. C) Recognition of the middle G/C pair. D) Recognition of the outer G/C pair.

In the proposed model of SfiI, the spatial configuration of the catalytic residues is typical for an active site architecture conserved among PD-DxK nucleases. We predict that the active site of SfiI comprises residues: E55, D79, D100 and K102, which superimpose well on the catalytic residues of BglI: E87, D116, D142 and K144, respectively (Figure 4). We predict that the DNA-binding mode of SfiI will be very similar to that of BglI, with the side chains of residues S210 and R218 (homologs of D268 and R277 in BglI) involved in the recognition of the inner C/G base pair (Figure 5a), backbone oxygen and the side chain of K208 (a homolog of K266 in BglI) recognizing the middle C/G base pair (Figure 5b), and the side chain of R220 (a homolog of R279) recognizing the G of the middle G/C base pair (Figure 5c). The specificity of SfiI towards the outer G/C base pair, not discriminated by BglI, can be explained by the development of new contacts made by residues from a divergent loop adjacent to the REase active site and comprising residues 104–110 of SfiI and 146–155 of BglI. In BglI, D150 makes specific contacts to the middle C/G base pair and the G/C base pair [30], however this residue is not conserved in SfiI (Figure 1). Instead, we predict that the changes of the loop length and the amino acid substitutions lead to a different conformation of the corresponding loop in SfiI, which allows R109 (not present in BglI) to make a specific contact to the G of the outer G/C base pair (Figure 5d). Other residues from the same loop, such as K107 may also contribute to the specific sequence recognition by SfiI by making contacts to either of the two G/C base pairs. It is noteworthy that according to our model of SfiI, the majority of specific contacts are achieved by three Arg residues (R109, R218, and R220). These predictions can be tested by site-directed mutagenesis of the respective residues to Ala and testing whether the mutant proteins are proficient in DNA cleavage and/or binding.

Interestingly, our model suggests that SfiI lacks the counterpart of a loop corresponding to aa 63–80 in BglI used by this enzyme to interact with the target site from the minor groove side. Thus, SfiI appears to recognize its target solely from the major groove side and to use fewer specific contacts than BglI to recognize its cognate site. This suggests that SfiI may be an easier target for the engineering of REases with new sequence specificities.

The dimerization interface of SfiI is comparable to that of BglI. We predict that the following residues may be important for monomer-monomer interactions: Q59, Y60, E63, E66, R73, F74, G76 and that mutating them to change the volume of the side chain (for instance G76R) or introducing (or reversing) the charge (E63R, E66R, R73D, F74R) could disrupt the formation of the SfiI dimer and destroy the REase activity.

### Prediction of the dimer-dimer interaction surface in the SfiI tetramer

**Figure 6:**
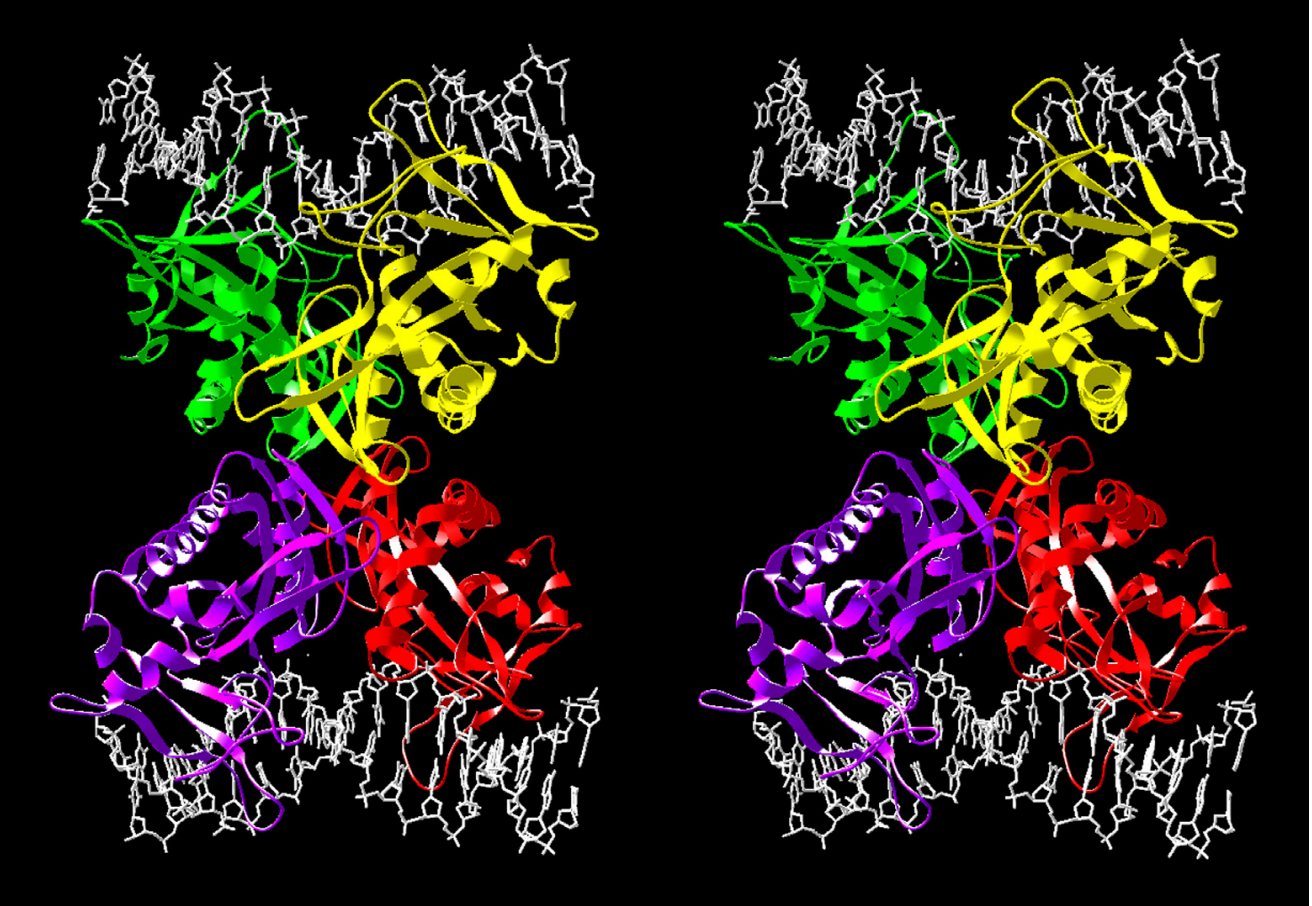
**Putative structure of the SfiI tetramer. **Individual subunits are shown in yellow, green, red, and magenta. The two DNA substrates are shown in white.

SfiI is a Type IIF enzyme, i.e. a tetramer that binds simultaneously to two recognition sites and cleaves both sites concertedly [21], while BglI is an orthodox IIP enzyme, i.e. a dimer that acts on single sites [38]. Therefore, the crystal structure of BglI cannot be used to model the tetrameric structure of SfiI. To date, the only Type IIF enzymes, for which crystal structures have been solved, are NgoMIV [39], Cfr10I [40] and Bse634I [41], which are all relatively closely related to each other and exhibit similar mode of interactions between two dimers within the tetramer. These enzymes, however, even at the level of a dimer exhibit a completely different arrangement of monomers, compatible with the generation of 5' overhangs 4 nt long (compared to 3' overhangs 3 nt long in the case of SfiI and BglI). Therefore, it is impossible to obtain a meaningful superposition of the BglI or SfiI dimer onto any pair of subunits in the NgoMIV, Cfr10I or Bse634I tetramer. However, it is tempting to speculate that SfiI may tetramerize in a similar manner to these enzymes, i.e. to use surface regions on the opposite sites of the molecule to the protein-DNA and protein-protein binding. A highly speculative model of SfiI tetramer obtained by manual docking of two dimers is shown in Figure 6. Based on this model, we predict that the dimer-dimer interface will be composed mostly of hydrophobic and polar residues (and very few charged ones), involve the following segments of the amino-acid sequence, corresponding to loops on the surface of the dimer: 26–34, 67–69, and 86–93. The putative dimer-dimer interactions involve contacts between hydrophobic regions (aa 86–93 from different subunits) as well as hydrophilic ones (aa 26–34). It is possible that the C-terminal region of the SfiI sequence, which could not be modeled (aa 241–269) may also participates in tetramerization.

## Conclusions

### Implications for the evolutionary history of different (sub) Types of REases

Comparative analysis of nucleases from the PD-DxK superfamily suggests that they can be classified into two remotely related lineages: α (EcoRI-like) and β (EcoRV-like) [7-9]. It was proposed that extant REases evolved independently from non-specific or structure-specific nucleases from both lineages (review: [5]). Interestingly, the phylogenetic tree of the PD-DxK superfamily revealed intriguing cases of convergent evolution. So far, it was found that Type IIE enzymes that bind two copies of the recognition site (the actual target of cleavage and the non-cleaved allosteric effector), evolved independently at least three times: EcoRII is an α-lineage member that apparently evolved from IIP enzymes similar to SsoII or PspGI by acquisition of an N-terminal effector-binding domain [12,42]. NaeI is a β-lineage member remotely related to IIP enzymes EcoRV and HincII that acquired a C-terminal effector-binding domain unrelated to that of EcoRII [7]. Finally, Sau3AI is a β-lineage member that apparently evolved by a duplication of a catalytic domain closely related to a DNA repair enzyme MutH, followed by the loss of catalytic residues in the C-terminal domain, thereby adapted to function as an effector-binding domain [14,15]. Another type of evidence for convergent evolution is provided by the finding that the specificity for the GATC sequence appeared independently in the α-lineage (MboI and its close homologs [5,13]) and in the β-lineage (Sau3AI and its close homologs [14,15]).

Our results strongly suggest that the archetypal Type IIF enzyme SfiI is closely related to a β-lineage member, an "orthodox" Type IIP REase BglI. Another well-characterized group of Type IIF REases comprises α-lineage members for which crystal structures were solved (NgoMIV, Cfr10I, Bse643I) [39-41]. Thus, our analysis provides evidence that the ability of Type IIF REases to tetramerize and cut two target sites in a concerted manner was developed independently at least two times in the evolution of the PD-DxK superfamily. Different Type IIF REases appear to have evolved independently from the simplest, "orthodox" Type IIP enzymes, like previously found for Type IIE REases. It was previously demonstrated that deletion of the effector-binding domain converts the Type IIE REase EcoRII to function as a Type IIP enzyme [43]. However, tetramerization seems to be important for the catalytic activity of the Type IIF enzyme Cfr10I, since the DNA cleavage activity of the dimeric W220A mutant of this REase is <0.1% of that of the wild-type enzyme [44]. In the absence of a high resolution co-crystal structure of SfiI in complex with DNA, our model will serve as a convenient platform to study sequence-structure-function relationships in this enzyme. In particular, it will facilitate the mutagenesis of residues potentially involved in tetramerization, dimerization, DNA-binding and catalysis.

## Methods

### Structure prediction

Sequence searches of the non-redundant (nr) database and of the putative translations from finished and unfinished microbial genomes were carried out at the NCBI using PSI-BLAST [45]. Secondary structure prediction and tertiary fold-recognition was carried out via the GeneSilico meta-server gateway [29]. Secondary structure prediction was predicted using PSIPRED [46], PROFsec [47], PROF [48], SABLE [49], JNET [50], JUFO [51], and SAM-T02 [33]. Solvent accessibility for the individual residues was predicted with SABLE [49] and JPRED [52]. The fold-recognition analysis (attempt to match the query sequence to known protein structures) was carried out using FFAS03 [53], SAM-T02 [33], 3DPSSM [34], INBGU [32], FUGUE [31], mGENTHREADER [54], and SPARKS [55]. Fold-recognition alignments reported by these methods were compared, evaluated, and ranked by the Pcons server [36].

### Homology modeling

The alignments between the sequence of SfiI and the structures of selected templates (members of the fold identified by Pcons) were used as a starting point for modeling of the SfiI tertiary structure using the "FRankenstein's Monster" approach [37], comprising cycles of model building by MODELLER [56], evaluation by VERIFY3D [57] via the COLORADO3D server [58], realignment in poorly scored regions and merging of best scoring fragments. The positions of predicted catalytic residues and secondary structure elements were used as spatial restraints. This strategy has previously helped us to build accurate, experimentally validated models of other REases, such as SsoII [11], PspGI [12], MboI [13], and KpnI [19].

## List of abbreviations

aa, amino acid(s); bp, base pair(s); nt, nucleotide; e, expectation; REase, restriction endonuclease; MTase, methyltransferase; ORF, product of an open reading frame, RM, restriction-modification;

## Authors' contributions

JMB carried out the fold-recognition analysis for SfiI, built the preliminary models, drafted the manuscript and coordinated the whole study. AC built the final models and identified functionally important residues. KS participated in interpretation of the data and writing the manuscript. All authors have read and accepted the final version of the manuscript.
